# Role of a new acellular dermal matrix in a multistep combined treatment of dermatofibrosarcoma protuberans of the lumbar region: a case report

**DOI:** 10.1186/s13256-021-02787-5

**Published:** 2021-04-20

**Authors:** Fedele Lembo, Liberato Roberto Cecchino, Domenico Parisi, Aurelio Portincasa

**Affiliations:** Unit of Reconstructive and Plastic Surgery, Azienda Ospedaliero Universitaria Ospedali Riuniti di Foggia, 71122 Foggia, Italy

**Keywords:** Dermatofibrosarcoma protuberans, Dermal regeneration template, Pelnac®, NPWT, Case report

## Abstract

**Background:**

Dermatofibrosarcoma protuberans (DFSP) is a rare skin fibroblastic tumor, with a high rate of recurrence. The treatment of DFSP is generally surgical, and wide local excision is the mainstay of surgical treatment. Therefore, complete assessment of all surgical margins is fundamental before definitive reconstruction. The reconstruction is a challenge for plastic surgeons, especially in particular anatomical areas (for aesthetic or functional problems) or in patients who are not candidates for more complex surgical treatments. We describe an alternative approach for reconstructive treatment of the lumbar area after wide excision of DFSP (without fresh-frozen sections) in a young obese woman with a history of smoking, using a new type of acellular dermal matrix (ADM) in a combined management protocol. The benefits of ADM are numerous: immediate wound closure and prevention of infections and excessive drying; minimal donor site morbidity; and good functional and aesthetic outcomes. Moreover, it is a temporary cover while the anatomical specimen is histologically analyzed, without donor site morbidity or prevention of any future surgery (if the margins are not tumor-free) or radiotherapy.

**Case presentation:**

In October 2019, a 34-year old obese Caucasian Woman with a history of smoking came to our institute for a multinodular growing polypoid mass in her lumbar region. An incisional biopsy diagnosed DFSP. The patient underwent proper staging. A wide local excision with 3 cm clinically healthy tissue margins down to the muscle fascia was performed and the defect was repaired using a combined approach with a new artificial bilaminar dermal template (Pelnac®, Gunze Ltd., Osaka, Japan) and a negative-pressure wound therapy system (V.A.C.®, KCI, San Antonio, USA). After the final histological examination revealed tumor-free margins, a split-thickness graft was harvested from the right gluteus and fixed to the new derma with negative-pressure wound therapy. Postoperative radiotherapy was not necessary. After 15 days, the wound had healed without complications, with satisfactory aesthetic outcome and with no limitation of back motion or pain. After 6 months of follow-up, the patient was free from disease.

**Conclusions:**

This is the first reported case of Pelnac® use in DFSP reconstruction of the lumbar region. We believe that the multistep approach described herein may be a good alternative approach in selected patients with wide resections in particular anatomical areas, especially when frozen sections (with Mohs micrographic surgery) are not available.

## Background

Dermatofibrosarcoma protuberans (DFSP) is a rare skin fibroblastic tumor, first described by Taylor in 1890, and named by Hoffman [1, 2].

This low-grade soft tissue sarcoma occurs at an annual rate of 0.3–0.5 cases/million persons. It typically occurs between the third and the sixth decade of life, with no male/female difference in rates (nearly 1:1). Genetic studies have identified a chromosomal error with the production of an altered protein (Collagen type 1 alpha 1 - platelet derived growth factor beta) that stimulates cell growth [3–6].

Clinically it is characterized by the following [3, 4, 7–11]:

- Silent clinical course (by slow growth)
- Variable lesion presentation (plaque-like or firm lesion with irregular borders or multinodular; with variable color from brownish to red, and size from 0.5 to more than 12 cm)
- common localization on the trunk, followed by the extremities; tendency to grow in areas exposed to trauma (such as burns, tattoo, X-ray)
- High tendency for local recurrence due to frequent infiltration of deeper structures
- Rare tendency to metastasize

The diagnosis is made by biopsy for histological examination.

The treatment of DFSP is generally surgical. Because this sarcoma grows deeply and laterally in subclinical mode, the challenge is to completely remove it with appropriate margin width. In the literature, we found that wide local excision is the mainstay of surgical treatment. However, there is no consensus on the safety margins; in fact, they range from 1 to 5 cm [12–16]. European consensus-based guidelines recommend 3 cm margins, excising the deep fascia to improve disease-free survival [17]. The role of fresh-frozen sections for evaluating tumor-free surgical margins is fundamental, but Mohs surgery is not available everywhere.

Local recurrence rates vary widely by as much as 40%; thus, follow-up every 6 months for 5 years is advised, then yearly for an additional 5 years. The prognosis is excellent, with a 5-year survival rate of 99% in cases of complete excision [18–21].

Complete assessment of all surgical margins is fundamental before definitive reconstruction, so meticulous preoperative planning and multiple surgical reconstructive procedure options are recommended for success [22–24]. Reconstruction is a challenge for plastic surgeons, especially in particular anatomical areas (for aesthetic or functional problems) or in patients who are not candidates for more complex surgical treatments.

We describe an alternative approach for reconstructive treatment of the lumbar area after wide excision of DFSP (without fresh-frozen sections) in a young obese woman with a history of smoking, using a new type of an acellular dermal matrix (ADM) in a combined management protocol.

## Case presentation

**Fig. 1:**
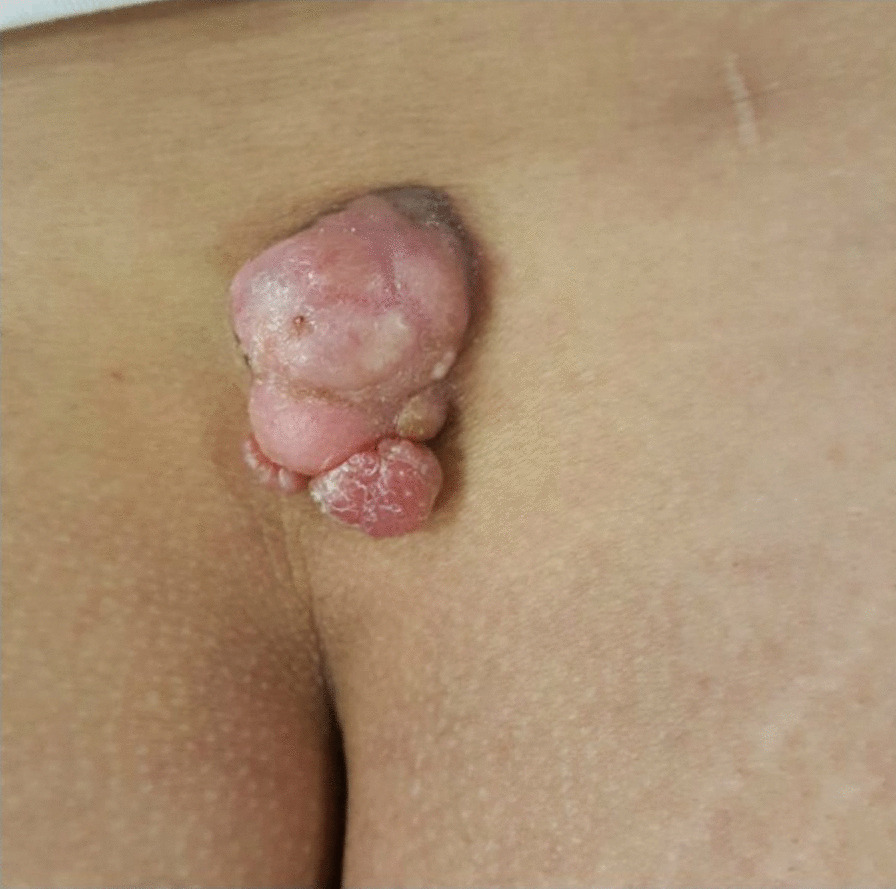
Preoperative view

In October 2019, a 34-year-old Caucasian woman came to the our institution for a multinodular growing polypoid mass in her lumbar region (Fig. 1), present for an unknown period of time. The patient was obese (Body Mass Index (BMI): 32.5) and a smoker; the remaining medical history was unremarkable.

An incisional biopsy diagnosed a DFSP. The patient underwent proper staging before surgery with head, chest, and abdominal computed tomography (CT) that showed absence of metastasis (T1a, N0, M0; stage 1A, according to the American Joint Committee on Cancer [AJCC] criteria).

**Fig. 2:**
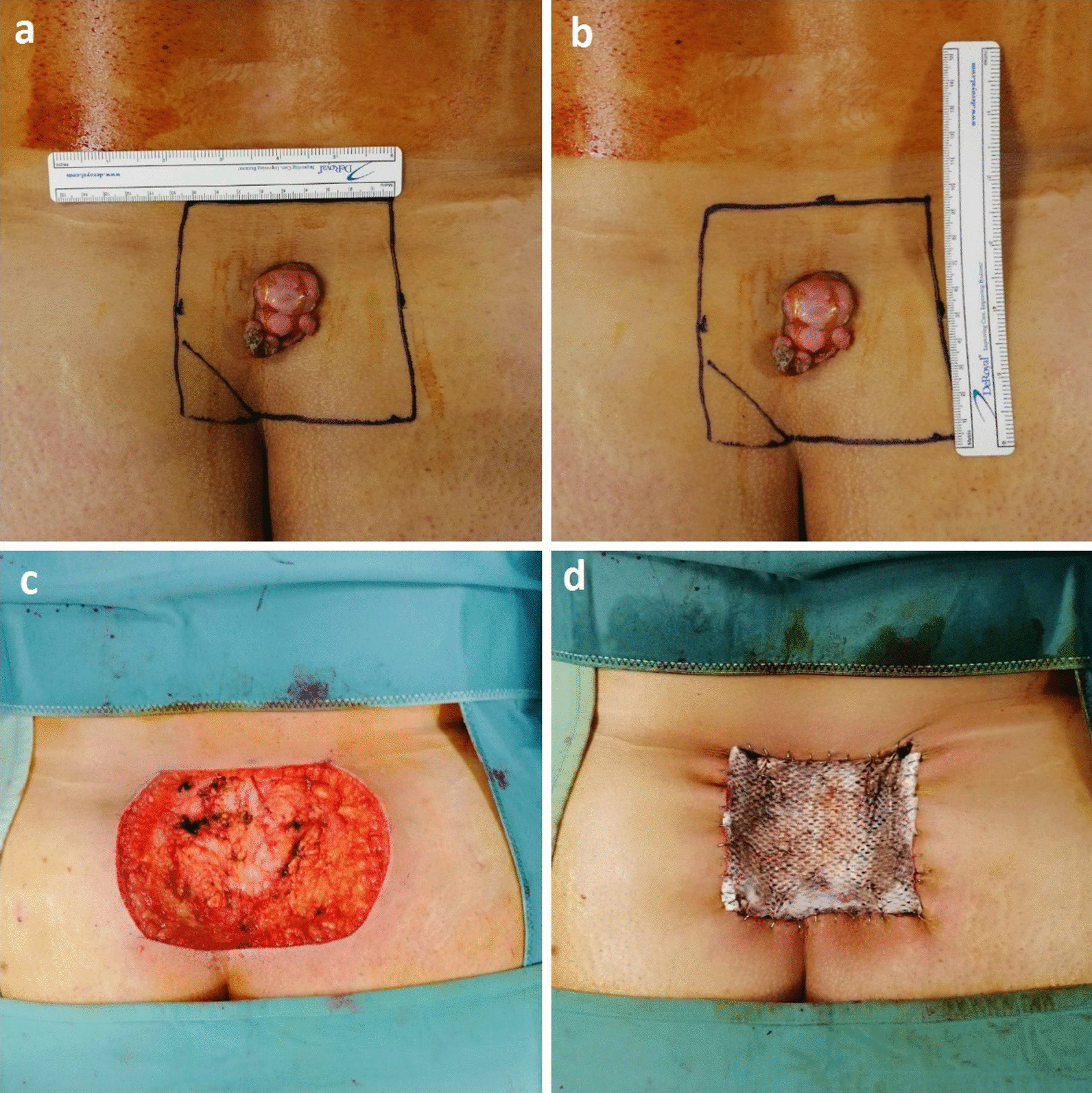
Intraoperative view of the surgical planning (**a**, **b**), the defect following tumor excision (**c**), and repair with the artificial dermal template Pelnac^®^ (**d**)

Therefore, a wide local excision was performed with 3 cm clinically healthy tissue margins down to the muscle fascia. The histopathological specimen measured 9 × 9.5 cm and included skin, subcutaneous tissue, and muscle fascia. Fresh-frozen sections were not available. The defect was repaired using a combined approach with a new artificial bilaminar dermal matrix (Pelnac^®^, Gunze Ltd., Osaka, Japan) and a negative-pressure wound therapy (NPWT) system (V.A.C.^®^, KCI, San Antonio, USA) (Fig. 2). The dermal regenerative template was secured on the wound with multiple staples, and the NPWT system (at 100 mmHg continuous negative pressure) was applied directly over the fenestrated silicon layer of the dermal matrix. The dressing was changed twice a week for 15 days. After the final histological examination revealed tumor-free margins, a split-thickness graft (STSG) was harvested from the right gluteus (because it is a covered area and it allows a unique operative position and medication) and fixed to the new derma with NPWT. Both surgical procedures were performed with local anesthesia, with good compliance by the patient. Postoperative radiotherapy was not necessary.

**Fig. 3:**
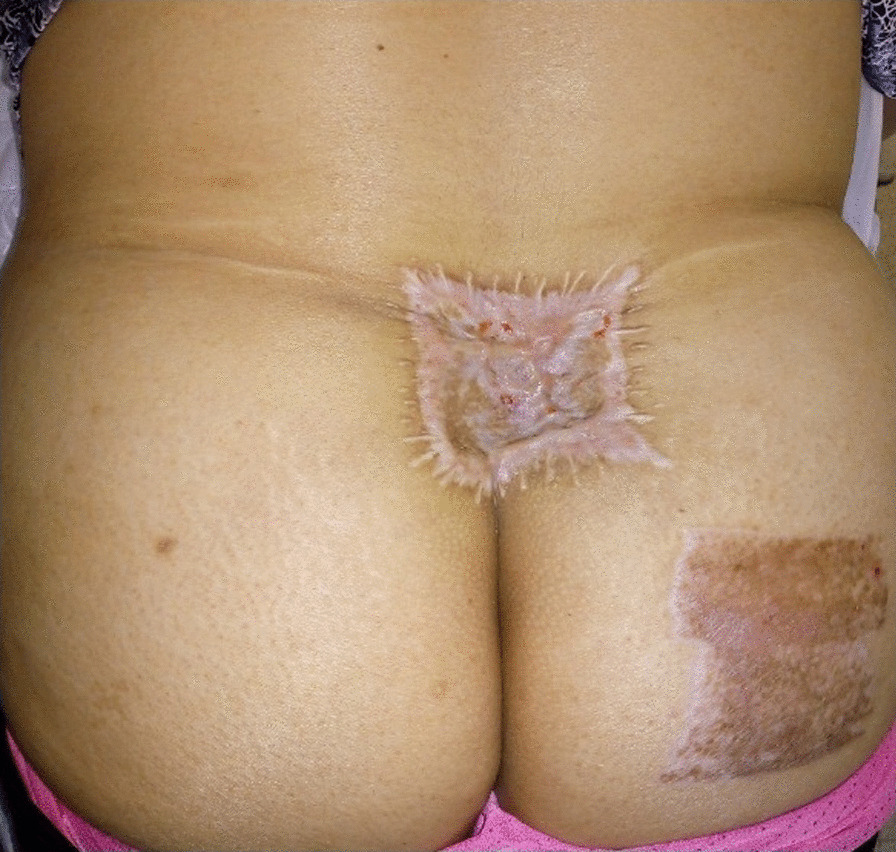
View of surgical outcome at 6 months postoperatively

At 15-day follow-up, the wound had healed without complications or adverse events, with satisfactory aesthetic outcome and without limitation of back motion or pain. After 6 months of follow-up, the patient was free from disease, with excellent quality of life (Fig. 3), and was satisfied with the treatments received.

## Discussion

The high recurrence rate of DFSP is the most important problem in surgical management; wide excision with margins free from disease is the cornerstone of therapy.

In the current literature, we found no consensus on the exact safety margins in wide excision; in fact, they range from 1 to 5 cm, and vary according to the site. Therefore, preoperative planning has a central role in avoiding inappropriate resection (positive margins), recurrence, and poor reconstructive outcomes of excision [12–21].

The reconstructive options, excluding primary closure, are full- or split-thickness skin graft and local, pedicled, or free flaps. Skin graft may be a good option in large resections except in cases when bone, nerves, and/or tendons are exposed or if a particular anatomical area is treated (for aesthetic or functional reasons). The quality of aesthetic outcomes is also poor due to the lack of elasticity of the grafted skin. On the other hand, skin flaps are more effective in protecting the exposed anatomical structure and do not undergo retraction. However, reconstruction using composite flaps requires surgical experience and an adequate operative/care setting, cannot be used in the case of severe and multiple comorbidities, and has a variable failure rate. Moreover, if Mohs micrographic surgery is not available (as in our center), and the definitive evaluation shows that the resection margins are not tumor-free, flap reconstruction can lead to a very difficult secondary surgery.

Thus, the dermal substitute (ADM) may be a good alternative. A literature search revealed only one case series report of surgical management of DFSP of the head and neck, which used the Integra^®^ dermal regeneration template [24] in a multistep protocol.

The ADM used in our case has different characteristics in composition, strength, resistance to traction force, time of integration, and costs. Although Pelnac^®^ systems are widely used in trauma, burns, and post-oncologic reconstruction [25], to date there is no report available on their use for reconstruction of the lumbar region after DFSP wide resection, especially combined with NPWT.

Pelnac^®^ is an artificial dermis (dermal template) made of two layers: an atelocollagen sponge layer (3 mm), which serves as the scaffold for dermal regeneration, and an outer reinforced silicon film layer (also fenestrated), which is able to prevent any infection from the outside. After application on the surgical bed, the collagen matrix is replaced with dermis-like tissue after 2–3 weeks. When a light reddish dermis-like tissue has formed, the outer silicon layer is removed and an STSG is applied. Various reports in the literature show that this ADM provides the formation of a consistent and elastic neodermis, very similar to endogenous tissue, improving cosmetic outcome. Furthermore, unlike other dermal matrices, it provides effective coverage of avascular wound beds due to peripheral revascularization into the matrix [26].

The benefits of ADM are numerous: immediate closure of the wound and prevention of infections and excessive drying; minimal donor site morbidity (as with STSG); and good functional and aesthetic outcomes (improved elasticity of STSG). Moreover, it is a temporary cover while the anatomical specimen is histologically analyzed, without donor site morbidity or prevention of any future surgery (if the margins are not tumor-free) or radiotherapy. In fact, this conservative strategy allows clinicians to reserve other surgical options for a secondary reconstruction if final histological examination requires a new wide excision with positive margins.

In the case reported herein, we also applied NPWT. There is evidence in the literature that the use of NPWT with artificial dermis can improve the healing process [27]. NPWT, in fact, shortens the interval between coverage and integration, evacuation of serum and blood, reducing the risk of infection, and minimizing the shear forces. Moreover, NPWT can be applied on the skin graft, avoiding additional splinting and guaranteeing better adhesion between the graft and the wound, even in “difficult” body surfaces.

The advantages of this multistep combined approach are as follows:

-Improved intake of ADM and of skin graft, especially in concave areas such as the lumbar region
- Lower complication rate and minimal donor site morbidity
- Guarantee of secure temporary cover during the definitive evaluation of the resection margins (if Mohs surgery is not available), leaving the opportunity for further secondary excision

The main disadvantages are the costs of the devices, patient compliance, and the need for multiple procedures.

## Conclusions

This is the first reported case of Pelnac^®^ use in DFSP reconstruction of the lumbar region. We believe that the multistep approach described herein may be a good alternative in selected patients with wide resections in particular anatomical areas, especially when frozen sections (with Mohs micrographic surgery) are not available.

## Data Availability

Please contact the corresponding author for data requests.

## Abbreviations

DFSP: Dermatofibrosarcoma protuberans
CT: Computed tomography
AJCC: American Joint Committee on Cancer
NPWT: Negative-pressure wound therapy
ADM: Acellular dermal matrix
STSG: Split-thickness skin graft
